# Supplementary material for: Early Motor Cortex Connectivity and Neuronal Reactivity in Intracerebral Hemorrhage: A Continuous-Wave Functional Near-Infrared Spectroscopy Study
Source: Sensors (Basel). 2025 Oct 15;25(20):6377. doi: 10.3390/s25206377 (PMC12567763; doi:10.3390/s25206377)
Supplement: Supplementary file 1 [file sensors-25-06377-s001.zip › sensors-3864092-supplementary.pdf]

Supplementary Materials

Supplementary Table S1. Functional mapping of fNIRS channels to EEG and brodmann areas for motor-sensory analysis.

	fNIRS Channels	10-10 EEG Representation	Broadmann's Representation
0	s1-d1	C1-FC1	MOTOR
1	s1-d2	C1-CP1	SENSORY
2	s1-d3	C1-C3	SENSORY
3	s2-d1	FC3-FC1	PREMOTOR
4	s2-d3	C3-FC3	MOTOR
5	s2-d4	FC3-FC5	PREMOTOR
6	s3-d3	CP3-C3	SENSORY
7	s4-d3	C3-C5	SENSORY/AT
8	s5-d5	C2-FC2	MOTOR
9	s5-d6	C2-CP2	SENSORY
10	s5-d7	C2-C4	SENSORY
11	s6-d5	FC4-FC2	PREMOTOR
12	s6-d7	C4-FC4	MOTOR
13	s7-d7	C4-CP4	SENSORY

Supplementary Table S2. Parameters for raw signal filtering during data processing.

	Resting	Finger Tapping	Handgrip
Iterations	10	10	10
Lag	5	5	5
Influence	0.50	0.50	0.50
Threshold	1	3.5	5

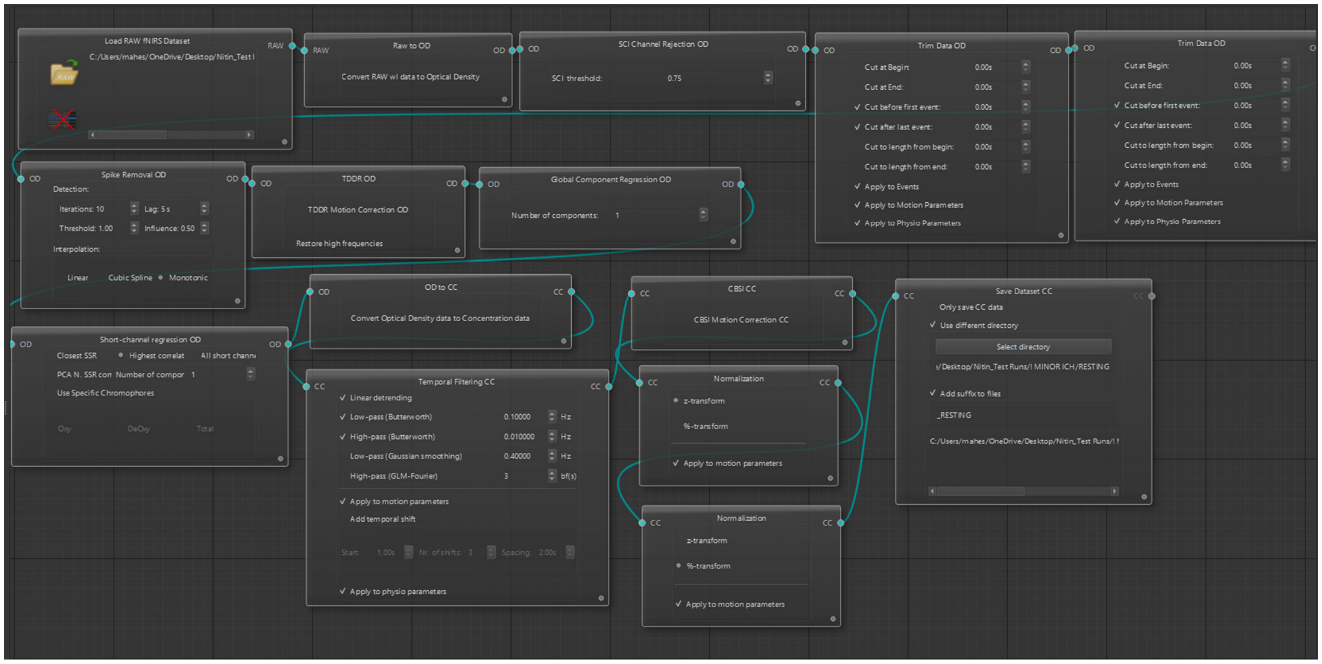

Supplementary Figure S1. Pre-processing pipeline for resting condition.

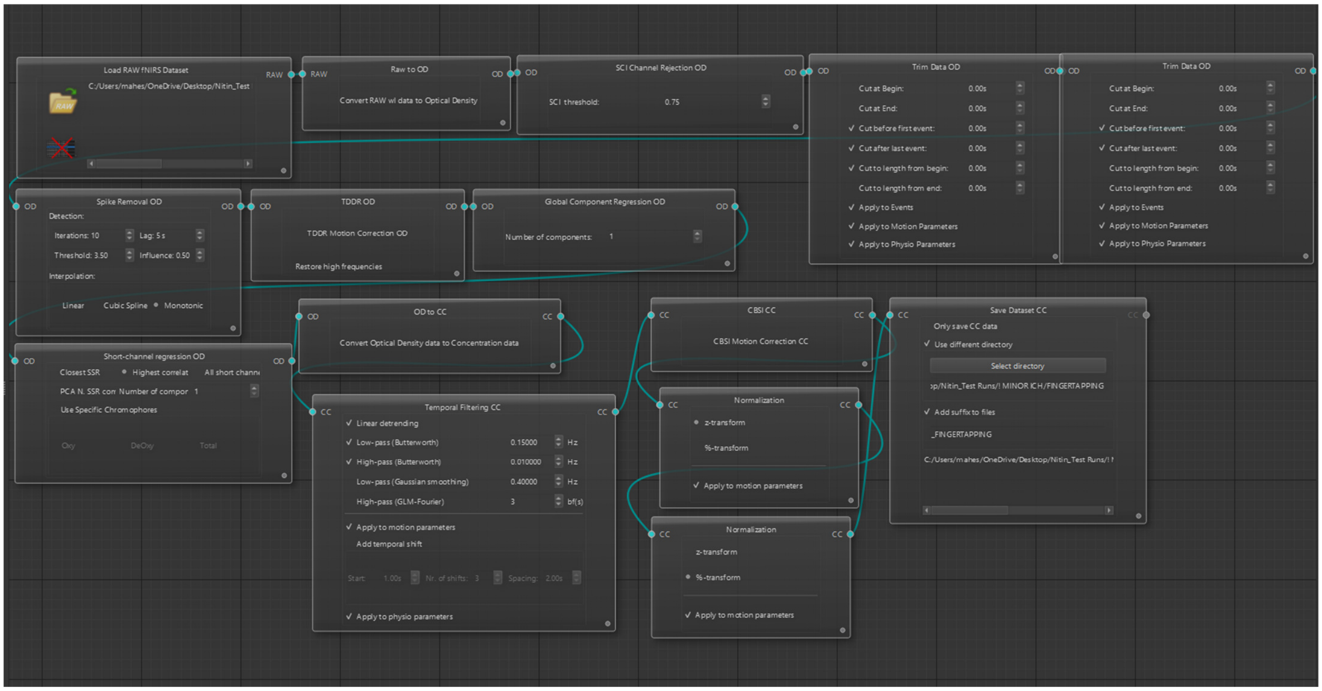

Supplementary Figure S2. Pre-processing pipeline for the finger-tapping condition.

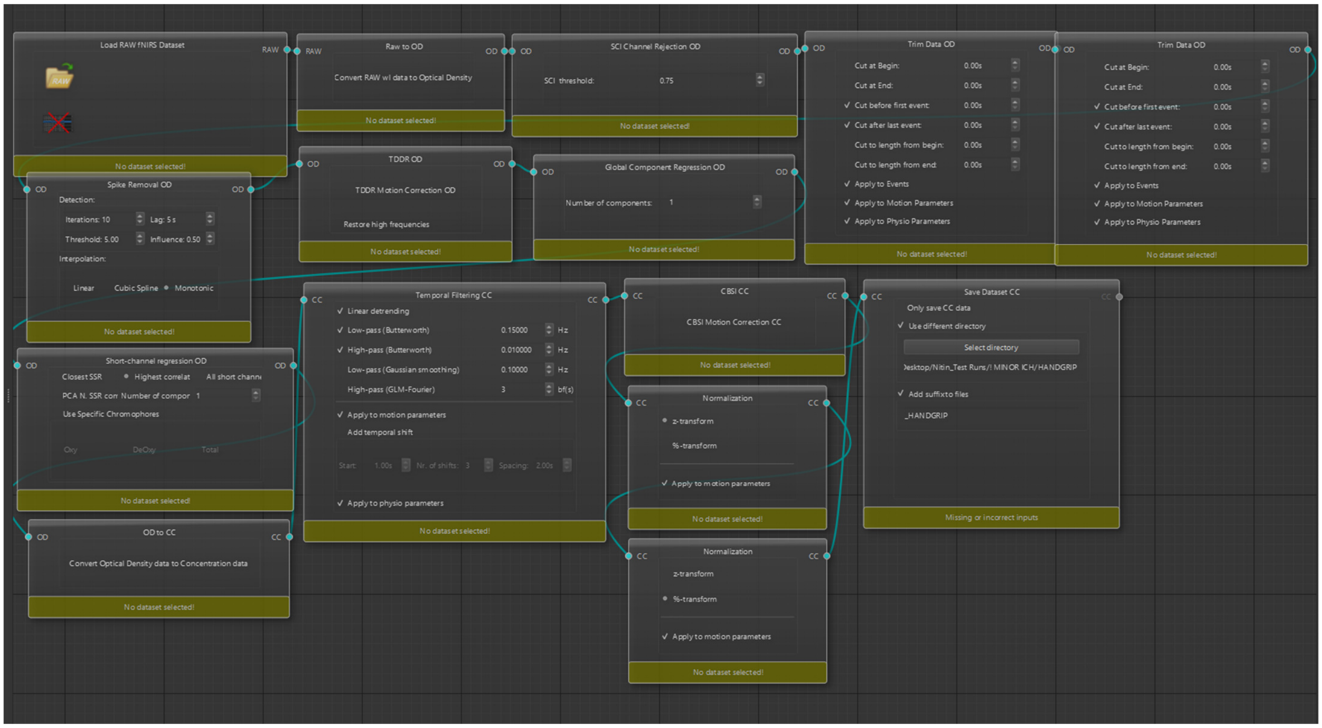

Supplementary Figure S3. Pre-processing pipeline for handgrip condition.
